# 4-Bromo-3-hy­droxy-3-(4-hy­droxy-2-oxo-2*H*-chromen-3-yl)indolin-2-one

**DOI:** 10.1107/S1600536811000213

**Published:** 2011-01-08

**Authors:** Song-Lei Zhu

**Affiliations:** a Department of Chemistry, Xuzhou Medical College, Xuzhou 221004, People’s Republic of China

## Abstract

In the mol­ecule of the title compound, C_17_H_10_BrNO_5_, the indoline system and the attached coumarin ring are each essentially planar with maximum deviations of 0.074 (2) and 0.062 (2) Å, respectively. The dihedral angle between them is 85.09 (3)°. In the crystal, all heteroatoms (except for the coumarin oxo O atoms) are involved in intra- and inter­molecular hydrogen bonds. An intra­molecular O—H⋯O hydrogen bond occurs. In the crystal, mol­ecules are linked through O—H⋯O, N—H⋯O and C—H⋯O contacts, forming a complex three-dimensional structure.

## Related literature

For general background to indoles and their biological activity, see: Da-Silva *et al.* (2001 ▶); Joshi & Chand (1982 ▶). Coumarin and its derivatives are important in the perfume, cosmetic and pharmaceutical industries, see: Soine (1964 ▶). For the synthesis of indole and coumarin derivatives in water, see: Zhu (2008 ▶).
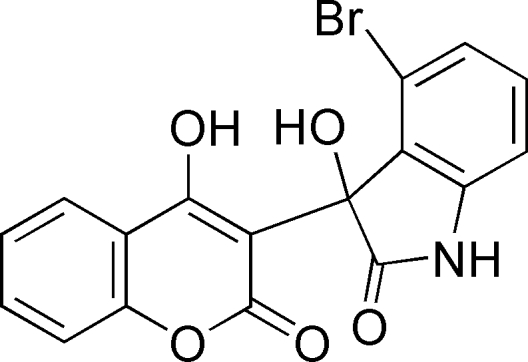


## Experimental

### 

#### Crystal data

C_17_H_10_BrNO_5_
*M* *_r_* = 388.17
Monoclinic, 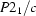

*a* = 11.358 (3) Å
*b* = 13.428 (3) Å
*c* = 10.360 (2) Å
β = 113.307 (3)°
*V* = 1451.1 (5) Å^3^
*Z* = 4
Mo *K*α radiation
μ = 2.86 mm^−1^
*T* = 153 K
0.78 × 0.36 × 0.35 mm

#### Data collection

Rigaku Mercury diffractometer
Absorption correction: multi-scan (*REQAB*; Jacobson, 1998 ▶) *T*_min_ = 0.173, *T*_max_ = 0.366
13755 measured reflections
2655 independent reflections
2544 reflections with *I* > 2σ(*I*)
*R*_int_ = 0.023

#### Refinement

*R*[*F* ^2^ > 2σ(*F* ^2^)] = 0.024
*wR*(*F* ^2^) = 0.059
*S* = 1.08
2655 reflections
220 parameters
H-atom parameters constrained
Δρ_max_ = 0.34 e Å^−3^
Δρ_min_ = −0.42 e Å^−3^

### 

Data collection: *CrystalClear* (Rigaku/MSC, 2001 ▶); cell refinement: *CrystalClear*; data reduction: *CrystalStructure* (Rigaku/MSC, 2004 ▶); program(s) used to solve structure: *SHELXS97* (Sheldrick, 2008 ▶); program(s) used to refine structure: *SHELXL97* (Sheldrick, 2008 ▶); molecular graphics: *ORTEPII* (Johnson, 1976 ▶) and *PLATON* (Spek, 2009 ▶); software used to prepare material for publication: *SHELXL97* and *PLATON* (Spek, 2009 ▶).

## Supplementary Material

Crystal structure: contains datablocks global, I. DOI: 10.1107/S1600536811000213/bh2329sup1.cif

Structure factors: contains datablocks I. DOI: 10.1107/S1600536811000213/bh2329Isup2.hkl

Additional supplementary materials:  crystallographic information; 3D view; checkCIF report

## Figures and Tables

**Table 1:** Hydrogen-bond geometry (Å, °)

*D*—H⋯*A*	*D*—H	H⋯*A*	*D*⋯*A*	*D*—H⋯*A*
O1—H1⋯O2^i^	0.84	1.98	2.7672 (17)	155
O5—H5⋯O1	0.84	1.81	2.5486 (19)	145
N1—H1*A*⋯O4^ii^	0.88	2.16	2.940 (2)	148
C7—H7⋯O2^iii^	0.95	2.49	3.429 (2)	172
C8—H8⋯O1^iii^	0.95	2.61	3.217 (2)	122

Symmetry codes: (i) 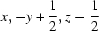; (ii) 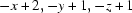; (iii) 
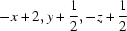
.

**Fig. 1.**
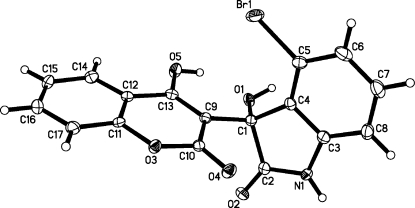
The molecular structure of the title molecule, with the atom-numbering scheme. Displacement ellipsoids are drawn at the 50% probability level.

**Fig. 2.**
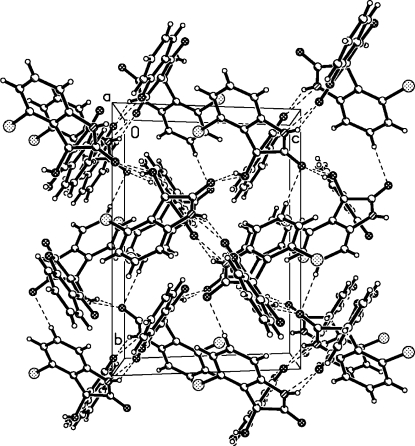
A packing diagram of the title compound. Hydrogen bonds are shown as dashed lines.

## supplementary crystallographic information

### Comment

The indole nucleus is a well known heterocycle (Da-Silva *et al.*, 2001). Compounds carrying the indole moiety exhibit antibacterial and fungicidal activities (Joshi & Chand, 1982). Coumarin and its derivatives are natural compounds and are also important chemicals in the perfume, cosmetic and pharmaceutical industries (Soine, 1964). As our interest in the synthesis of heterocyclic compounds, guided by the observation that the presence of two or more different heterocyclic moieties in a single molecule often remarkably enhances the biocidal profile, we investigated a simple and green protocol for the synthesis of indole and coumarin derivatives in water (Zhu, 2008). We report herein the crystal structure of the title compound.

In the molecule (Fig. 1), the indole ring (C1···C4/N1/C5···C8) and the attached coumarin ring (C9···C17/O3), are both planar. The dihedral angle between them is 85.09 (3)°. In the crystal structure, intermolecular and intramolecular O—H···O, N—H···O and C—H···O hydrogen bonds (Table 1) link the molecules (Fig. 2), in which they may be effective in the stabilization of the structure.

### Experimental

The title compound was prepared by the reaction of 4-bromoisatin (4-bromoindole-2,3-dione, 2 mmol) and 4-hydroxy-2*H*-chromen-2-one (2 mmol) in water (10 ml). The reaction was catalyzed by TEBAC (triethylbenzylammonium chloride, 1 mmol). After stirring at 333 K for 3 h, the reaction mixture was cooled and washed with small amount of ethanol. The crude product was filtered and single crystals of the title compound were obtained from an ethanol solution by slow evaporation at room temperature (yield: 80%; m.p. 469–471 K). Spectroscopic analysis: IR (KBr, ν, cm^-1^): 3401, 3368, 3251, 3177, 2955, 1710, 1673, 1613, 1520, 1478, 1314, 1231, 1165, 1057, 922, 756, 612, 568. ^1^H-NMR (400 MHz, DMSO-*d*_6_): 9.88 (br s, 1H, NH), 7.84 (t, *J* = 7.2 Hz, 1H, ArH), 7.58-6.64 (m, 2H, ArH), 6.75-6.82 (m, 2H, ArH), 6.52- 6.59(m, 2H, ArH), 2.41(s, 1H, OH).

### Refinement

H atoms were positioned geometrically, with N—H = 0.88 Å (for NH), O—H = 0.84 Å (for OH) and C—H = 0.95 Å for aromatic H, respectively, and constrained to ride on their parent atoms with *U*_iso_(H) = 1.2 *U*_eq_(C, N, O).

### Crystal data

**Table:**

C_17_H_10_BrNO_5_	*F*(000) = 776
*M**_r_* = 388.17	*D*_x_ = 1.777 Mg m^−^^3^
Monoclinic, *P*2_1_/*c*	Melting point = 469–471 K
Hall symbol: -P 2ybc	Mo *K*α radiation, λ = 0.71070 Å
*a* = 11.358 (3) Å	Cell parameters from 5845 reflections
*b* = 13.428 (3) Å	θ = 3.0–25.3°
*c* = 10.360 (2) Å	µ = 2.86 mm^−^^1^
β = 113.307 (3)°	*T* = 153 K
*V* = 1451.1 (5) Å^3^	Block, colourless
*Z* = 4	0.78 × 0.36 × 0.35 mm

### Data collection

**Table:**

Rigaku Mercury diffractometer	2655 independent reflections
Radiation source: fine-focus sealed tube	2544 reflections with *I* > 2σ(*I*)
graphite	*R*_int_ = 0.023
Detector resolution: 7.31 pixels mm^-1^	θ_max_ = 25.4°, θ_min_ = 3.0°
ω scans	*h* = −13→11
Absorption correction: multi-scan (REQAB; Jacobson, 1998)	*k* = −16→16
*T*_min_ = 0.173, *T*_max_ = 0.366	*l* = −12→12
13755 measured reflections	

### Refinement

**Table:**

Refinement on *F*^2^	Primary atom site location: structure-invariant direct methods
Least-squares matrix: full	Secondary atom site location: difference Fourier map
*R*[*F*^2^ > 2σ(*F*^2^)] = 0.024	Hydrogen site location: inferred from neighbouring sites
*wR*(*F*^2^) = 0.059	H-atom parameters constrained
*S* = 1.08	*w* = 1/[σ^2^(*F*_o_^2^) + (0.0278*P*)^2^ + 1.0659*P*] where *P* = (*F*_o_^2^ + 2*F*_c_^2^)/3
2655 reflections	(Δ/σ)_max_ = 0.001
220 parameters	Δρ_max_ = 0.34 e Å^−^^3^
0 restraints	Δρ_min_ = −0.42 e Å^−^^3^
0 constraints	

### Fractional atomic coordinates and isotropic or equivalent isotropic displacement parameters (Å^2^)

**Table:**

	*x*	*y*	*z*	*U*_iso_*/*U*_eq_	
Br1	0.618858 (19)	0.424530 (16)	−0.05888 (2)	0.02468 (8)	
O1	0.81967 (12)	0.24579 (9)	0.19817 (13)	0.0150 (3)	
H1	0.8602	0.2544	0.1466	0.023*	
O2	0.93364 (12)	0.28564 (10)	0.50987 (13)	0.0165 (3)	
O3	0.55747 (12)	0.43528 (9)	0.37776 (13)	0.0147 (3)	
O4	0.75296 (12)	0.48622 (9)	0.41805 (13)	0.0170 (3)	
O5	0.58573 (13)	0.20063 (10)	0.13673 (14)	0.0195 (3)	
H5	0.6572	0.1978	0.1306	0.029*	
N1	1.01146 (14)	0.40717 (11)	0.40768 (16)	0.0144 (3)	
H1A	1.0875	0.4158	0.4757	0.017*	
C1	0.81079 (17)	0.33888 (13)	0.26299 (18)	0.0122 (4)	
C2	0.92400 (17)	0.33989 (13)	0.41181 (19)	0.0130 (4)	
C3	0.96558 (18)	0.46179 (14)	0.28075 (19)	0.0149 (4)	
C4	0.84479 (18)	0.42644 (13)	0.19296 (19)	0.0131 (4)	
C5	0.78170 (18)	0.47149 (14)	0.06449 (19)	0.0169 (4)	
C6	0.8382 (2)	0.55024 (15)	0.0235 (2)	0.0223 (4)	
H6	0.7943	0.5817	−0.0648	0.027*	
C7	0.9589 (2)	0.58249 (15)	0.1123 (2)	0.0239 (5)	
H7	0.9975	0.6359	0.0832	0.029*	
C8	1.02526 (19)	0.53885 (15)	0.2429 (2)	0.0196 (4)	
H8	1.1081	0.5613	0.3034	0.023*	
C9	0.68273 (17)	0.34335 (13)	0.27623 (18)	0.0124 (4)	
C10	0.66978 (17)	0.42446 (13)	0.35962 (19)	0.0124 (4)	
C11	0.45996 (17)	0.36647 (14)	0.32443 (18)	0.0135 (4)	
C12	0.46973 (17)	0.28643 (14)	0.24491 (18)	0.0139 (4)	
C13	0.58467 (17)	0.27702 (14)	0.21777 (18)	0.0136 (4)	
C14	0.37095 (18)	0.21509 (15)	0.20037 (19)	0.0176 (4)	
H14	0.3758	0.1597	0.1457	0.021*	
C15	0.26732 (18)	0.22596 (16)	0.2364 (2)	0.0203 (4)	
H15	0.2014	0.1772	0.2084	0.024*	
C16	0.25902 (18)	0.30829 (16)	0.3140 (2)	0.0214 (4)	
H16	0.1864	0.3156	0.3370	0.026*	
C17	0.35425 (18)	0.37954 (15)	0.35806 (19)	0.0178 (4)	
H17	0.3476	0.4360	0.4100	0.021*	

### Atomic displacement parameters (Å^2^)

**Table:**

	*U*^11^	*U*^22^	*U*^33^	*U*^12^	*U*^13^	*U*^23^
Br1	0.02298 (13)	0.02838 (13)	0.01533 (12)	−0.00237 (8)	−0.00025 (9)	0.00135 (8)
O1	0.0179 (7)	0.0140 (7)	0.0178 (7)	−0.0007 (5)	0.0120 (5)	−0.0031 (5)
O2	0.0181 (7)	0.0185 (7)	0.0146 (6)	0.0034 (5)	0.0084 (5)	0.0029 (5)
O3	0.0115 (6)	0.0166 (7)	0.0166 (7)	−0.0005 (5)	0.0061 (5)	−0.0045 (5)
O4	0.0156 (7)	0.0151 (7)	0.0192 (7)	−0.0024 (5)	0.0058 (5)	−0.0056 (5)
O5	0.0166 (7)	0.0213 (7)	0.0231 (7)	−0.0047 (6)	0.0106 (6)	−0.0118 (6)
N1	0.0099 (7)	0.0185 (8)	0.0131 (8)	−0.0012 (6)	0.0028 (6)	−0.0004 (6)
C1	0.0130 (9)	0.0116 (9)	0.0125 (9)	−0.0004 (7)	0.0057 (7)	−0.0017 (7)
C2	0.0125 (9)	0.0130 (9)	0.0157 (9)	0.0032 (7)	0.0078 (7)	−0.0013 (7)
C3	0.0159 (9)	0.0154 (9)	0.0153 (9)	0.0010 (7)	0.0082 (7)	−0.0021 (8)
C4	0.0153 (9)	0.0122 (9)	0.0143 (9)	−0.0005 (7)	0.0085 (8)	−0.0019 (7)
C5	0.0192 (10)	0.0174 (10)	0.0139 (9)	0.0005 (8)	0.0064 (8)	−0.0024 (8)
C6	0.0326 (12)	0.0192 (10)	0.0158 (10)	0.0000 (9)	0.0101 (9)	0.0038 (8)
C7	0.0338 (12)	0.0179 (10)	0.0256 (11)	−0.0060 (9)	0.0177 (10)	0.0002 (8)
C8	0.0204 (10)	0.0182 (10)	0.0226 (10)	−0.0061 (8)	0.0113 (8)	−0.0035 (8)
C9	0.0121 (9)	0.0136 (9)	0.0112 (9)	0.0009 (7)	0.0045 (7)	−0.0002 (7)
C10	0.0114 (9)	0.0141 (9)	0.0110 (9)	0.0008 (7)	0.0038 (7)	0.0018 (7)
C11	0.0111 (9)	0.0177 (9)	0.0089 (8)	−0.0008 (7)	0.0010 (7)	0.0011 (7)
C12	0.0128 (9)	0.0173 (9)	0.0102 (8)	0.0001 (7)	0.0031 (7)	0.0021 (7)
C13	0.0164 (9)	0.0137 (9)	0.0103 (8)	0.0012 (7)	0.0048 (7)	−0.0003 (7)
C14	0.0176 (9)	0.0185 (10)	0.0155 (9)	−0.0021 (8)	0.0051 (8)	−0.0023 (8)
C15	0.0129 (9)	0.0272 (11)	0.0191 (10)	−0.0068 (8)	0.0045 (8)	−0.0005 (8)
C16	0.0138 (9)	0.0339 (12)	0.0181 (10)	0.0004 (8)	0.0080 (8)	0.0008 (9)
C17	0.0153 (9)	0.0238 (10)	0.0143 (9)	0.0033 (8)	0.0056 (8)	−0.0016 (8)

### Geometric parameters (Å, °)

**Table:**

Br1—C5	1.8925 (19)	C6—C7	1.384 (3)
O1—C1	1.441 (2)	C6—H6	0.9500
O1—H1	0.8400	C7—C8	1.392 (3)
O2—C2	1.219 (2)	C7—H7	0.9500
O3—C10	1.369 (2)	C8—H8	0.9500
O3—C11	1.379 (2)	C9—C13	1.366 (3)
O4—C10	1.222 (2)	C9—C10	1.434 (2)
O5—C13	1.329 (2)	C11—C12	1.385 (3)
O5—H5	0.8400	C11—C17	1.387 (3)
N1—C2	1.356 (2)	C12—C14	1.407 (3)
N1—C3	1.413 (2)	C12—C13	1.446 (3)
N1—H1A	0.8800	C14—C15	1.376 (3)
C1—C4	1.510 (2)	C14—H14	0.9500
C1—C9	1.515 (2)	C15—C16	1.392 (3)
C1—C2	1.569 (2)	C15—H15	0.9500
C3—C8	1.376 (3)	C16—C17	1.379 (3)
C3—C4	1.395 (3)	C16—H16	0.9500
C4—C5	1.377 (3)	C17—H17	0.9500
C5—C6	1.388 (3)		
			
C1—O1—H1	109.5	C3—C8—C7	117.08 (18)
C10—O3—C11	121.22 (14)	C3—C8—H8	121.5
C13—O5—H5	109.5	C7—C8—H8	121.5
C2—N1—C3	111.72 (15)	C13—C9—C10	120.16 (16)
C2—N1—H1A	124.1	C13—C9—C1	125.27 (16)
C3—N1—H1A	124.1	C10—C9—C1	114.56 (15)
O1—C1—C4	111.91 (14)	O4—C10—O3	116.09 (16)
O1—C1—C9	108.86 (14)	O4—C10—C9	124.78 (17)
C4—C1—C9	116.74 (15)	O3—C10—C9	119.13 (15)
O1—C1—C2	106.50 (14)	O3—C11—C12	121.17 (16)
C4—C1—C2	101.50 (14)	O3—C11—C17	116.90 (16)
C9—C1—C2	110.71 (14)	C12—C11—C17	121.91 (17)
O2—C2—N1	126.80 (17)	C11—C12—C14	118.83 (16)
O2—C2—C1	125.59 (16)	C11—C12—C13	118.33 (16)
N1—C2—C1	107.49 (15)	C14—C12—C13	122.75 (17)
C8—C3—C4	122.56 (18)	O5—C13—C9	125.02 (16)
C8—C3—N1	127.83 (17)	O5—C13—C12	115.15 (16)
C4—C3—N1	109.61 (16)	C9—C13—C12	119.82 (16)
C5—C4—C3	118.82 (17)	C15—C14—C12	119.70 (18)
C5—C4—C1	132.24 (17)	C15—C14—H14	120.2
C3—C4—C1	108.91 (16)	C12—C14—H14	120.2
C4—C5—C6	120.25 (18)	C14—C15—C16	120.10 (18)
C4—C5—Br1	120.15 (14)	C14—C15—H15	120.0
C6—C5—Br1	119.59 (15)	C16—C15—H15	120.0
C7—C6—C5	119.43 (19)	C17—C16—C15	121.25 (18)
C7—C6—H6	120.3	C17—C16—H16	119.4
C5—C6—H6	120.3	C15—C16—H16	119.4
C6—C7—C8	121.85 (19)	C16—C17—C11	118.19 (18)
C6—C7—H7	119.1	C16—C17—H17	120.9
C8—C7—H7	119.1	C11—C17—H17	120.9
			
C3—N1—C2—O2	−176.25 (17)	C2—C1—C9—C13	−125.59 (19)
C3—N1—C2—C1	7.53 (19)	O1—C1—C9—C10	170.24 (14)
O1—C1—C2—O2	−67.8 (2)	C4—C1—C9—C10	−61.9 (2)
C4—C1—C2—O2	175.02 (17)	C2—C1—C9—C10	53.5 (2)
C9—C1—C2—O2	50.4 (2)	C11—O3—C10—O4	−175.77 (15)
O1—C1—C2—N1	108.53 (15)	C11—O3—C10—C9	3.9 (2)
C4—C1—C2—N1	−8.69 (17)	C13—C9—C10—O4	178.59 (17)
C9—C1—C2—N1	−133.29 (15)	C1—C9—C10—O4	−0.5 (3)
C2—N1—C3—C8	176.71 (18)	C13—C9—C10—O3	−1.0 (3)
C2—N1—C3—C4	−3.0 (2)	C1—C9—C10—O3	179.82 (15)
C8—C3—C4—C5	−1.2 (3)	C10—O3—C11—C12	−3.1 (2)
N1—C3—C4—C5	178.55 (16)	C10—O3—C11—C17	174.87 (16)
C8—C3—C4—C1	177.16 (17)	O3—C11—C12—C14	176.13 (16)
N1—C3—C4—C1	−3.1 (2)	C17—C11—C12—C14	−1.7 (3)
O1—C1—C4—C5	71.8 (2)	O3—C11—C12—C13	−0.6 (3)
C9—C1—C4—C5	−54.5 (3)	C17—C11—C12—C13	−178.41 (17)
C2—C1—C4—C5	−174.98 (19)	C10—C9—C13—O5	178.61 (17)
O1—C1—C4—C3	−106.26 (17)	C1—C9—C13—O5	−2.4 (3)
C9—C1—C4—C3	127.40 (17)	C10—C9—C13—C12	−2.5 (3)
C2—C1—C4—C3	6.96 (18)	C1—C9—C13—C12	176.51 (16)
C3—C4—C5—C6	0.4 (3)	C11—C12—C13—O5	−177.70 (16)
C1—C4—C5—C6	−177.55 (18)	C14—C12—C13—O5	5.8 (3)
C3—C4—C5—Br1	179.05 (13)	C11—C12—C13—C9	3.3 (3)
C1—C4—C5—Br1	1.1 (3)	C14—C12—C13—C9	−173.22 (17)
C4—C5—C6—C7	0.6 (3)	C11—C12—C14—C15	−0.1 (3)
Br1—C5—C6—C7	−178.13 (15)	C13—C12—C14—C15	176.45 (17)
C5—C6—C7—C8	−0.7 (3)	C12—C14—C15—C16	1.4 (3)
C4—C3—C8—C7	1.0 (3)	C14—C15—C16—C17	−1.0 (3)
N1—C3—C8—C7	−178.66 (18)	C15—C16—C17—C11	−0.8 (3)
C6—C7—C8—C3	−0.1 (3)	O3—C11—C17—C16	−175.81 (16)
O1—C1—C9—C13	−8.8 (2)	C12—C11—C17—C16	2.1 (3)
C4—C1—C9—C13	119.0 (2)		

### Hydrogen-bond geometry (Å, °)

**Table:**

*D*—H···*A*	*D*—H	H···*A*	*D*···*A*	*D*—H···*A*
O1—H1···O2^i^	0.84	1.98	2.7672 (17)	155
O5—H5···O1	0.84	1.81	2.5486 (19)	145
N1—H1A···O4^ii^	0.88	2.16	2.940 (2)	148
C7—H7···O2^iii^	0.95	2.49	3.429 (2)	172
C8—H8···O1^iii^	0.95	2.61	3.217 (2)	122

Symmetry codes: (i) *x*, −*y*+1/2, *z*−1/2; (ii) −*x*+2, −*y*+1, −*z*+1; (iii) −*x*+2, *y*+1/2, −*z*+1/2.
